# Synergism between the Synthetic Antibacterial and Antibiofilm Peptide (SAAP)-148 and Halicin

**DOI:** 10.3390/antibiotics11050673

**Published:** 2022-05-17

**Authors:** Miriam E. van Gent, Tanny J. K. van der Reijden, Patrick R. Lennard, Adriëtte W. de Visser, Bep Schonkeren-Ravensbergen, Natasja Dolezal, Robert A. Cordfunke, Jan Wouter Drijfhout, Peter H. Nibbering

**Affiliations:** 1 Department of Infectious Diseases, Leiden University Medical Center, 2300 RC Leiden, The Netherlands; t.j.k.van_der_reijden@lumc.nl (T.J.K.v.d.R.); p.r.lennard@sms.ed.ac.uk (P.R.L.); a.w.de_visser@lumc.nl (A.W.d.V.); e.schonkeren@lumc.nl (B.S.-R.); p.h.nibbering@lumc.nl (P.H.N.); 2 Department of Pulmonology, Leiden University Medical Center, 2300 RC Leiden, The Netherlands; 3 Center for Inflammation Research, Queen’s Medical Research Institute, University of Edinburgh, Edinburgh EH16 4TJ, UK; 4 Department of Immunology, Leiden University Medical Center, 2300 RC Leiden, The Netherlands; n.dolezal@lumc.nl (N.D.); r.a.cordfunke@lumc.nl (R.A.C.); j.w.drijfhout@lumc.nl (J.W.D.)

**Keywords:** SAAP-148, halicin, biofilm, synergy, antimicrobial resistance, bladder infection, skin wound infection

## Abstract

Recently, using a deep learning approach, the novel antibiotic halicin was discovered. We compared the antibacterial activities of two novel bactericidal antimicrobial agents, i.e., the synthetic antibacterial and antibiofilm peptide (SAAP)-148 with this antibiotic halicin. Results revealed that SAAP-148 was more effective than halicin in killing planktonic bacteria of antimicrobial-resistant (AMR) *Escherichia coli, Acinetobacter baumannii* and *Staphylococcus aureus*, especially in biologically relevant media, such as plasma and urine, and in 3D human infection models. Surprisingly, SAAP-148 and halicin were equally effective against these bacteria residing in immature and mature biofilms. As their modes of action differ, potential favorable interactions between SAAP-148 and halicin were investigated. For some specific strains of AMR *E. coli* and *S. aureus* synergism between these agents was observed, whereas for other strains, additive interactions were noted. These favorable interactions were confirmed for AMR *E. coli* in a 3D human bladder infection model and AMR *S. aureus* in a 3D human epidermal infection model. Together, combinations of these two novel antimicrobial agents hold promise as an innovative treatment for infections not effectively treatable with current antibiotics.

## 1. Introduction

Effective antibiotics are essential to our society as they are widely used for the treatment and prevention of bacterial infections. In addition, they are a prerequisite for medical innovations, such as transplantations, experimental surgery and advanced tumor treatments. Already by the 1950s, most classes of current antibiotics had been discovered, mainly through the screening of secondary metabolites from environmental microbes for bactericidal activities [1,2]. Since the 1970s, a wide variety of analogues and derivatives of these antibiotics with improved activity and pharmacokinetic properties and (slightly) less resistance has been developed. However, so far, almost no antibiotics with a mode of action different from the current antibiotics have been developed. Different approaches have been used in attempts to discover novel antimicrobial agents. Synthetic biology has focused, for example, on polyketide synthases and non-ribosomally produced peptide synthases. This approach has been successful in the production of new antibiotics, such as macrolides and tetracycline antibiotics, and novel peptides with bactericidal activities [2]. Recently, a novel class of ribosomally synthesized and post-translationally modified peptides (RiPPs) was discovered using machine learning algorithms to predict novel RiPP precursors [3].

Unfortunately, (ab)use of antibiotics in humans and animals has contributed to the worldwide emergence of antimicrobial-resistant (AMR) bacterial strains [4], which may lead to a scenario in which simple infections, such as bladder infections, respiratory tract infections and skin wound infections, pose a major threat to public health. In addition, biofilm and persister formation by these pathogens further challenge the efficacy of antibiotics [5,6]. Together, the increasing failure of antibiotics highlights the need for innovative therapeutic agents. Consequently, current research focused on naturally occurring antimicrobial peptides (AMPs) from an array of species, ranging from plants to mammals, including humans. These AMPs have been isolated from biological sources and/or identified by mining their genomes. Libraries of modified peptides have been synthesized and tested for their ability to combat bacterial infections, including infections caused by antimicrobial resistant micro-organisms. Synthetic antimicrobial and antibiofilm peptides (SAAPs), such as SAAP-148, are bactericidal agents that are highly effective against (AMR) bacterial infections in vitro and in animal models [7,8]. SAAP-148 quickly induces bacterial cell death upon interaction with the bacterial plasma membrane, resulting in membrane destabilization, permeability and leakage [7]. Importantly, another peptide from the SAAP family, i.e., P60.4Ac, proved successful in the treatment of chronic otitis media in therapy-resistant patients [9], indicating the potential of SAAPs.

Over the last few decades, the use of computer-assisted tools has increased dramatically in the search for novel AMPs and antibiotics [10,11]. For instance, using the Drug Repurposing Hub, a deep learning model recently identified halicin as a new antibiotic [12]. Additional experiments indicated halicin to be bactericidal in in vitro experiments and in mice. The mode of action of halicin was ascribed to its ability to dissipate the transmembrane ΔpH potential of the proton motive force. Likely, halicin interferes with the electrochemical transmembrane gradient via prior complexation with iron, which results in reduced viability and eventually the death of bacterial cells. In addition, the formulation of this antibiotic into electron-spun fibers, which can potentially be used as wound dressing, maintained biological activity against strains of *Escherichia coli*, *Staphylococcus aureus* and *Acinetobacter baumannii,* while completely releasing halicin in two hours [13].

In light of the above, the present study compared the in vitro antibacterial and cytotoxic activities of SAAP-148 and halicin. Since the majority of clinical infections are biofilm-associated infections, the antibiofilm activities of both agents against 24-h-immature and 7-days-mature biofilms were determined. Combined treatment with these two bactericidal agents, which have a different mode of action, can have attractive advantages. Therefore, this study aimed at identifying possible synergistic effects between SAAP-148 and halicin. Here, we demonstrate that SAAP-148 and halicin act synergistically against strains of *E. coli* and *S. aureus*. These favorable interactions were validated in relevant 3D models for *E. coli* bladder infections and *S. aureus* skin wound infections.

## 2. Results

### 2.1. Bactericidal Activities of SAAP-148 and Halicin

Comparison of the bactericidal activities of SAAP-148 and halicin towards log-phase bacteria upon 4-h exposure in different physiological environments revealed that SAAP-148 was more effective than halicin in killing AMR *E. coli*, *A. baumannii* and *S. aureus* in PBS (Table 1). In addition, the bactericidal activity of halicin was reduced to a greater extent by factors present in biological fluids, like urine and plasma, than that of SAAP-148. Both agents were effective against stationary-phase bacteria (data not shown), which indicates that the bactericidal activity of halicin, like SAAP-148, is not dependent on the metabolic activity of the bacteria.

Comparison of the antibacterial activities of SAAP-148 and halicin against planktonic bacteria upon 4-h exposure in PBS, 50% human plasma or 50% urine (in the case of *E. coli*). Results are expressed as the LC_99.9_, i.e., the lowest concentration at which 99.9% of the bacteria are killed. Results are the medians (bold) and ranges of three independent experiments; each performed in duplicate.

### 2.2. Anti-Biofilm Activities of SAAP-148 and Halicin

Next, the ability of SAAP-148 and halicin to eradicate bacteria in both 24-h-immature and 7-days-mature biofilms of AMR *E. coli*, *A. baumannii* and *S. aureus* upon 4-h and 24-h exposure was assessed microbiologically. Results revealed that SAAP-148 and halicin dose-dependently reduced bacterial counts in immature and mature biofilms. In general, SAAP-148 and halicin were similarly effective against bacteria in immature and mature biofilms (Table 2). However, high concentrations of SAAP-148, but not halicin, completely eradicated all bacteria in immature and mature biofilms. Importantly, 24-h exposure was more effective than 4-h exposure for halicin, except for exposure to 24-h immature *A. baumannii* biofilms.

24-h-immature and 7-days-mature biofilms were exposed for 4 h and 24 h to increasing concentrations of SAAP-148 and halicin in PBS. Anti-biofilm activities are expressed as the BIC_99_, i.e., the lowest concentration of the agents resulting in a 99% reduction in bacterial counts compared to the control. Results are the medians (bold) and ranges of three independent experiments; each performed in duplicate.

### 2.3. Hemolytic and Cytotoxic Activities of SAAP-148 and Halicin

**Figure 1:**
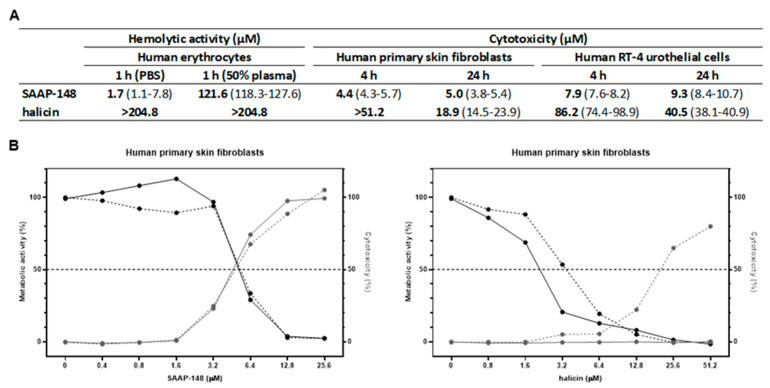
**Hemolytic and cytotoxic activities of SAAP-148 and halicin.** (**A**) Values presented are cytotoxicity values of (i) 2% human erythrocytes in PBS or 50% plasma after 1 h exposure to SAAP-148 or halicin, (ii) a monolayer of human primary skin fibroblasts in DMEM medium with 0.5% human serum after 4 h and 24 h exposure to SAAP-148 or halicin and (iii) a monolayer of RT-4 urothelial cells in McCoy’s medium with 10% fetal calf serum after 4 h and 24 h exposure to SAAP-148 or halicin. Results are depicted as the medians (bold) and ranges of the IC_50_, i.e., the calculated concentration of the agents resulting in 50% cytotoxicity, of three independent experiments performed in triplicate. (**B**) Metabolic activity and cytotoxicity (determined by LDH release) of human primary skin fibroblasts upon exposure to SAAP-148 and halicin in DMEM medium with 0.5% human serum for 4 h (solid) and 24 h (dashed). Results are depicted as medians of three independent experiments performed in triplicate.

Furthermore, SAAP-148 and halicin were compared regarding their hemolytic activity and cytotoxicity against human primary skin fibroblasts and RT-4 urothelial cells. SAAP-148 dose-dependently induced cytotoxicity to human erythrocytes, skin fibroblasts and RT-4 cells. In contrast to SAAP-148, halicin did not show any hemolytic activity at concentrations up to 204.8 µM for 1 h in PBS or 50% plasma (Figure 1A). Importantly, 51.2 µM, 102.4 µM and 204.8 µM halicin in combination with SAAP-148 did not enhance the hemolytic activity of SAAP-148 in 50% plasma. Similarly, halicin was less cytotoxic for skin fibroblasts and RT-4 cells compared to SAAP-148 as determined by LDH release. Interestingly, cytotoxicity upon exposure to halicin increased with time, while cytotoxicity due to SAAP-148 remained unchanged. Surprisingly, skin fibroblasts displayed a greatly reduced metabolic activity, without significant LDH release upon exposure for 4 h to halicin (Figure 1B). In contrast, after 24 h of exposure to halicin, these cells displayed both reduced metabolic activity and enhanced LDH release. Together, these data indicate that SAAP-148 is more cytotoxic than halicin and that cytotoxicity of halicin, but not SAAP-148, increases with time of exposure. Furthermore, the hemolytic activity of SAAP-148 is not affected when SAAP-148 is combined with halicin.

### 2.4. Favourable Interactions between SAAP-148 and Halicin towards Strains of AMR E. coli and S. aureus

**Figure 2:**
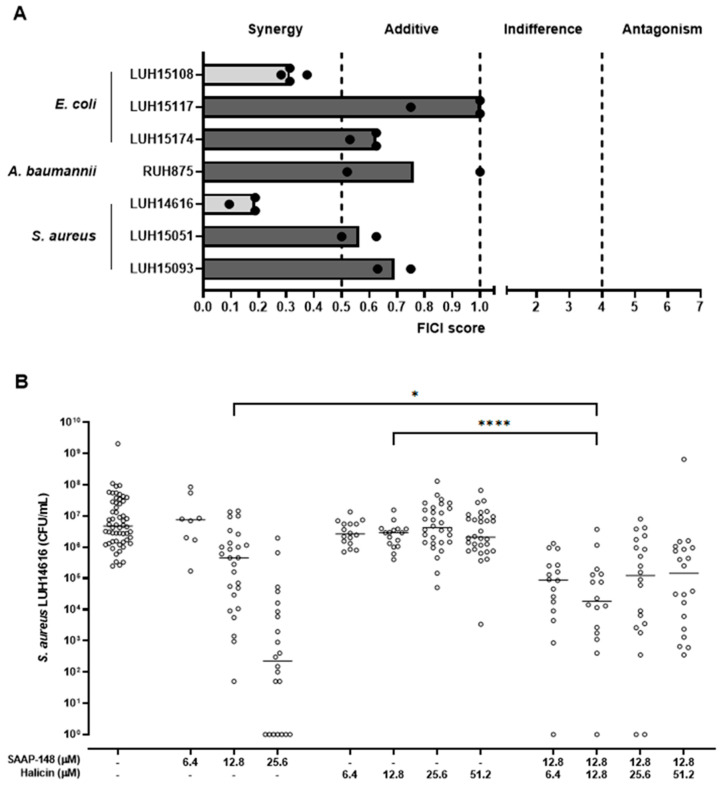
**Synergistic and additive interactions between SAAP-148 and halicin.** (**A**) fractional inhibitory concentration index (FICI) scores for exposure of planktonic cells of several bacterial strains to SAAP-148, halicin and combinations thereof. Interactions are considered synergistic or additive with a FICI score <0.5 or between 0.5 and 1, respectively, while interactions are considered indifferent or antagonistic with a FICI score between 1 and 4 or >4, respectively. Results are depicted as median and ranges of two to three (when additive effect) or three to four (when synergism) independent experiments. (**B**) Bacterial load of *S. aureus* (LUH14616) biofilms adhered to silicone disks after 4 h exposure to SAAP-148, halicin or combinations thereof. Results are shown as individual values and medians of at least two independent experiments performed in quadruplicate. Statistical differences between the two groups are depicted as * for *p* ≤ 0.1 and **** for *p* ≤ 0.0001 as calculated by the Mann-Whitney U-test.

As the modes of action of SAAP-148 and halicin differ [7,12], checkerboard assays were performed to investigate whether combinations of SAAP-148 and halicin act synergistically or exerted additive effects on planktonic AMR *E. coli*, *A. baumannii* and *S. aureus*. Results revealed synergy between SAAP-148 and halicin for *E. coli* strain LUH15108 and *S. aureus* strain LUH14616, with FICI scores of 0.3125 and 0.1875 respectively, while for other strains of *E. coli*, *A. baumannii* and *S. aureus* combinations of SAAP-148 and halicin acted in an additive fashion (Figure 2A). Next, possible favorable interactions between SAAP-148 and halicin were assessed on biofilm-residing bacteria attached to silicone disks, which mimic synthetic materials such as intravenous and urinary tract catheters. The results revealed a dose-dependent reduction of AMR *S. aureus* bacteria (LUH14616) after exposure to SAAP-148 or halicin, with SAAP-148 being more effective (Figure 2B). Exposure of the biofilms to 12.8 µM SAAP-148 in combination with 12.8 µM halicin resulted in a significantly stronger reduction of bacterial counts compared to single-agent exposure. However, at higher concentrations of halicin, no additive effects were observed. Together, this data illustrates that synergism between SAAP-148 and halicin is strain specific and that combinations of SAAP-148 and halicin are able to further reduce bacterial loads in AMR *S. aureus* biofilms attached to silicone disks compared to single-agent exposure, although complete eradication of bacteria was only observed in one of sixteen disks.

### 2.5. Effect of Combinations of SAAP-148 and Halicin on AMR E. coli in a 3D Human Bladder Model

**Figure 3:**
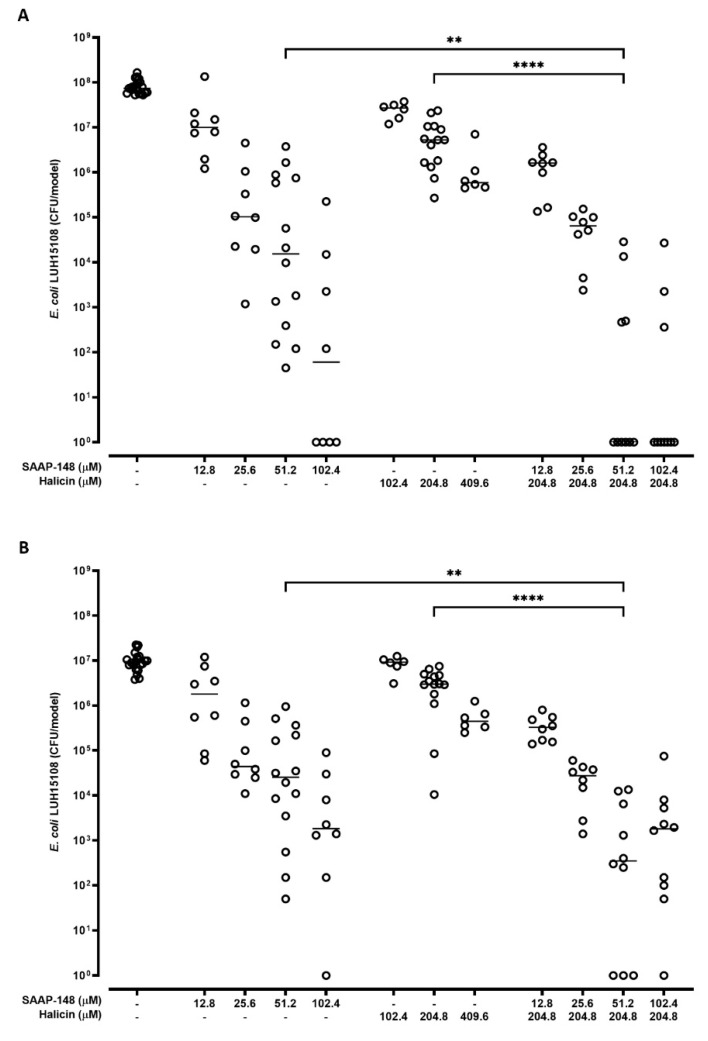
**Bactericidal effects of SAAP-148 and halicin and combinations thereof on *E. coli* in the luminal and cellular compartments of 3D bladder infection models.** Bacterial load of *E. coli* (LUH15108) in (**A**) the supernatant (luminal compartment) and (**B**) the model (cellular compartment) of 3D bladder models composed of RT-4 cells after 4 h exposure to SAAP-148, halicin or combinations thereof. Results are shown as individual values and medians of three to seven independent experiments performed in duplicate. Statistical differences between the two groups are depicted as ** for *p* ≤ 0.01 and **** for *p* ≤ 0.0001 as calculated by the Mann–Whitney U-test.

As combinations of SAAP-148 and halicin may be promising as a treatment for bladder infections, the efficacy of combinations of SAAP-148 and halicin was compared to that of these agents alone against AMR *E. coli* LUH15108 strain in a 3D-urothelial infection model. Results revealed a dose-dependent reduction of *E. coli* in both the luminal and the cellular compartment after exposure to SAAP-148 and less effectively to halicin (Figure 3). Interestingly, the combination of 51.2 µM SAAP-148 and 204.8 µM halicin was significantly more effective than these agents alone, indicating that in the presence of halicin lower concentrations of SAAP-148 were required to reduce the bacterial counts in the bladder lumen and tissue. At these concentrations of SAAP-148 and halicin the RT-4 cells of the bladder models maintained metabolically active (data not shown). Of note, 408.9 µM halicin did not further enhance these favorable interactions.

### 2.6. Effect of Combinations of SAAP-148 and Halicin on AMR S. aureus in a 3D Human Epidermal Model

**Figure 4:**
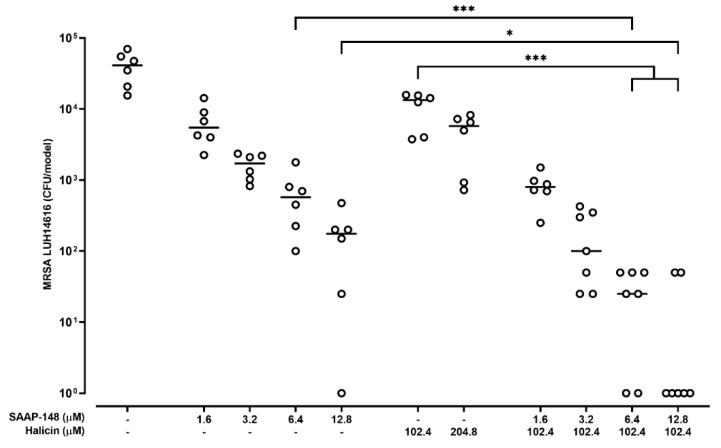
**Bactericidal effects of SAAP-148 and halicin and combinations thereof on *S. aureus* in the adherent fraction of 3D epidermal infection model.** Bacterial load of adherent bacteria of MRSA (LUH14616) infected 3D human epidermal models after 4-h exposure to SAAP-148, halicin or combinations thereof. Results are shown as individual values and medians of three independent experiments performed in duplicate or triplicate. Statistical differences between the two groups are depicted as * for *p* ≤ 0.1 and *** for *p* ≤ 0.001 as calculated by the Mann–Whitney U-test.

Lastly, the efficacy of SAAP-148 and halicin and combinations thereof were assessed against AMR *S. aureus* LUH14616 strain on 3D epidermal models. Results revealed a dose-dependent reduction of *S. aureus* bacteria adherent to the skin models (Figure 4) upon exposure to SAAP-148 and halicin, with SAAP-148 being more effective. Combinations of SAAP-148 and halicin were significantly more effective than these agents alone, with 12.8 µM of SAAP-148, in combination with 102.4 halicin, completely eradicating bacteria in most models. Similar results were obtained for SAAP-148 in combination with 204.8 µM halicin. At these concentrations of SAAP-148 and halicin, the Ker-CT cells of the skin models did not release LDH in the supernatant fraction (data not shown).

## 3. Discussion

The efficacy of antibiotics is increasingly jeopardized by the emergence of AMR strains and/or biofilm and persistence formation. Here, we compared the efficacy of two novel antimicrobial agents on AMR bacteria and such bacteria within biofilms in vitro and assessed possible favorable interactions between these agents. In addition, these in vitro results were validated in relevant 3D models, which mimic hard-to-treat bladder and skin wound infections.

A comparison of the antibacterial and antibiofilm activities of SAAP-148 and halicin on bacteria in vitro revealed that upon 4-h exposure, SAAP-148 is more effective than halicin against planktonic bacteria of three AMR bacterial strains, especially in biologically relevant media such as plasma or urine. As reported before, components in plasma and urine can reduce the antimicrobial activity of AMPs and antibiotics [14]. It should be mentioned that halicin is expected to increase activity upon longer exposure periods based on previous research by Stokes et al. [12], while SAAP-148 is effective within minutes and only marginally increases activity upon longer exposure periods [7]. In addition, both agents are effective against stationary-phase AMR *E. coli* and *S. aureus* bacteria. In agreement, Stokes et al. reported that halicin induces cell death of metabolically repressed *E. coli* [12]. These findings suggest that the bacterial target of halicin, like SAAP-148, is not associated with bacterial cell metabolism. Surprisingly, SAAP-148 and halicin were equally effective against bacteria residing in biofilm. A comparison of planktonic (LC_99.9_) and antibiofilm (BIC_99_) values upon 4 h of exposure revealed that biofilms require a 43- to 256-fold increased concentration of SAAP-148 to reach the BIC_99_, while only an 8- to 16-fold increased concentration of halicin is required. These results raise the question why halicin eradicates planktonic bacteria less effectively than SAAP-148, while it is equally effective against immature and mature biofilms. A possible explanation could be that halicin penetrates biofilms more efficiently than SAAP-148. The efficacy of SAAP-148 is hampered by electrostatic interactions with negatively charged polymers of the biofilm matrix [15]. In addition, halicin is much smaller than SAAP-148 and most likely diffuses more easily into the biofilm matrix than SAAP-148 [16,17]. This possible explanation could be tested using confocal microscopy, which allows imaging of biofilms treated with fluorescently labelled agents in a three-dimensional manner [18,19,20]. Another explanation could be that halicin, aside from its bactericidal activity, affects the gene expression of biofilm-residing bacteria, specifically of genes involved in quorum sensing and biofilm formation. As result bacteria will be released from the biofilm into the medium as single colonies and/or planktonic mode. To investigate this possibility, transcriptomics on halicin-exposed bacteria could be used to identify the upregulation of gene expression associated with bacterial biofilm formation, maintenance and quorum sensing [21,22,23,24,25]. Thirdly, halicin is an inhibitor of the c-Jun N-terminal kinase (IC_50_ = 0.7 µM; [26]) and was originally researched for the treatment of diabetes. A main difficulty in kinase inhibitor development is the nonspecific kinase activity of inhibitors targeting the kinase ATP-binding site, which is highly conserved across kinases [27]. Therefore, it cannot be excluded that halicin also inhibits bacterial kinases, e.g., histidine kinases are involved in the two-component signal transduction system (TCS) of bacteria [28] regulating biofilm formation and maintenance. It should be realized that bacterial histidine kinases do share their ATP-binding domain, which contains a Bergerat fold, with eukaryotic kinases [29]. This possibility of bacterial kinase inhibition may be investigated using activity-based protein profiling [30] to reveal potential bacterial kinase targets of halicin.

A comparison of the hemolytic and cytotoxic activities of SAAP-148 and halicin in vitro revealed that SAAP-148 was more cytotoxic than halicin, although the latter increased cytotoxicity upon longer exposure times, e.g., at 24 h. Increased cytotoxicity over time towards human cells was shown before for other antibiotics, such as vancomycin [31]. AMPs are more cytotoxic to human erythrocytes, where increased hydrophobicity of the AMP is positively correlated to cytotoxicity, while human cell lines are usually more tolerant towards these AMPs. In addition, the in vitro cytotoxicity of SAAP-148 and halicin was shown to be dependent on several factors, including extracellular micro-environment, cell type and organization of the cells in, e.g., 3D structures. Moreover, combinations of halicin and SAAP-148 did not affect the hemolytic activity of SAAP-148 in 50% plasma. Most importantly, the in vitro cytotoxicity very often cannot be translated into in vivo models [32]. Both SAAP-148 and halicin have been successful against infections in in vivo models [7,26,33]. Together, SAAP-148 is more effective and more cytotoxic than halicin. Nevertheless, a selectivity profile, where the agent of interest kills bacteria at lower concentrations than it induces cytotoxicity, is necessary. This target selectivity can be improved by either decreasing the cytotoxicity of the agent or by increasing its antibacterial activity. Cytotoxicity of AMPs and/or antibiotics can be reduced by using nanoscale drug delivery systems, such as liposomes, PLGA nanoparticles and hyaluronic-acid based nanogels (reviewed in van Gent et al. [34]). In addition, these drug delivery systems target intracellular bacteria, enhance the bioavailability of the agent at the site of infection and enhance penetration into the biofilm [34,35,36]. Alternatively, AMPs can be combined with other agents to circumvent adverse cytotoxicity by reducing the required concentration of peptide while maintaining the ability to eliminate bacteria.

As combination therapies have several advances over monotherapies, such as reduction of cytotoxic effects and less resistance development [37,38], we investigated possible favorable interactions between SAAP-148 and halicin. We report favorable interactions and even synergism between SAAP-148 and halicin against planktonic AMR *E. coli* and *S. aureus* bacteria. Previously, for both halicin and SAAP-148 synergistic effects have been described with classical antibiotics [12,39,40]. As clinical infections involve both planktonic and biofilm-associated bacteria, it is of relevance to test whether favorable interactions between SAAP-148 and halicin can also be observed for bacteria residing in biofilms. Although, SAAP-148 and halicin alone did reduce bacterial growth of AMR *S. aureus* biofilms grown on silicone disks, combinations of these agents reduced bacterial counts more than these agents alone. Of note, these combinations did not completely eliminate biofilm-residing bacteria. Importantly, the in vitro synergy results obtained in this study were validated in clinically relevant 3D models, including human bladder models infected with AMR *E. coli* and human epidermal models infected with AMR *S. aureus*. The antibacterial effect of both agents was confirmed in these models, again with SAAP-148 being more effective than halicin. Notably, favorable interactions between SAAP-148 and halicin were found in both 3D models, showing the clinical potential of combinatory treatment with SAAP-148 and halicin against bladder infections and skin wound infections. Of note, halicin could have additional potential in the treatment of diabetic wound infections. In type-two diabetic, insulin-insensitive mice, halicin treatment prior to insulin injection resulted in reduced blood glucose levels due to inhibition of the JNK pathway [26,33]. High glucose levels can affect wound healing and therefore halicin has the potential to allow faster wound healing [41,42].

As the development of resistant bacteria is emerging rapidly, new therapies approved for clinical use are highly needed. The main limitation of this study is that synergistic and/or additive combinations were only tested in vitro; however, these results were confirmed in 3D bladder and skin infection models. Of course, it is of most importance to include in vivo animal models in future research to reveal the true potential of combinatory treatment with SAAP-148 and halicin. Related to this, the safety of the use of halicin for the treatment of bacterial infections should be verified in future clinical studies, i.e., that halicin is not able to block the c-Jun N-terminal kinase in humans as an off-target. Interestingly, despite in vitro cytotoxicity of SAAP-148 and halicin reported in this study, both agents have been successful against infections in vivo [7,26,33], indicating that the tolerability of these agents in vivo may not always be reflected by the in vitro cytotoxicity values. In the case of urinary tract infections, intravascular application of antibiotics is relatively safe as a result of only short exposure to high concentrations of antibiotics [43]. In addition, minor toxicity towards the bladder epithelium might in fact aid the eradication of bacteria as exfoliation of outer epithelium cell layers is an effective process to reduce bacterial load during bladder infections [44]. Furthermore, administration of a combination of two diverse agents, as is the case with SAAP-148 and halicin, faces difficulties related to differences in pharmacokinetic and/or pharmacodynamic properties of the two agents. This limitation can perhaps be overcome by conjugating SAAP-148 with halicin [38,45]. Nevertheless, our in vitro findings show that combinations of SAAP-148 and halicin allow for the use of lower SAAP-148 concentrations while maintaining excellent antimicrobial activities and without affecting the hemolytic activity of SAAP-148. Taken together, combinations of SAAP-148 and halicin are promising as treatment for bladder and/or skin wound infections not effectively treatable with current antibiotics.

## 4. Materials and Methods

### 4.1. Antibacterial Agents

SAAP-148 (acetyl-LKRVWKRVFKLLKRYWRQLKKPVR-amide) was synthesized by Fmoc chemistry on an automated peptide synthesizer (Syro ll, MultiSyntech, Witten, Germany), as described previously [46]. The molecular mass of the peptide (3269.6 g/mol) was confirmed by mass spectrometry and purity amounted to >95%, as determined by ultra-high-performance liquid chromatography. Lyophilized peptide was stored at −20 °C until use. For the experiments, a stock solution of 5.12 mM SAAP-148 was prepared in Milli-Q and further diluted in PBS to obtain working solutions ≤ 204.8 µM. Halicin (SU 3327; 261.3 g/mol) was obtained from Tocris Bioscience (Bristol, UK). For experiments, halicin was dissolved in DMSO to a concentration of 51.2 mM and further diluted in PBS to obtain working solutions ≤ 204.8 µM.

### 4.2. Bacteria

The following strains were used in this study: antimicrobial-resistant (AMR) *A. baumannii* strain RUH875; MRSA strains LUH14616 (NCCB100829, AMR), LUH15051 (methicillin and mupirocin resistant) and LUH15093 (SAC042W/USA300); and *E. coli* strains LUH15108 (AMC1677, a pathogenic strain), LUH15117 (colistin resistant, ESKAPE panel (AMR)) and LUH15174 (SPA012, invasive strain, AMR). Bacteria were stored in glycerol at −80 °C until use. Prior to experiments, bacteria were cultured on blood agar plates overnight at 37 °C. Thereafter, bacteria were cultured to mid-log phase in tryptic soy broth (TSB) for 2.5 h under continuous rotation, centrifuged at 1000× *g* for 10 min, the broth was removed, and the bacteria were resuspended in the preferred medium to the required concentrations based on the optical density at 600 nm.

### 4.3. In Vitro Killing Assay

Mid-log phase bacteria were resuspended in phosphate-buffered saline (PBS; pH 7.4) to a concentration of 5 × 10^6^ CFU/mL. Subsequently, 30 μL of PBS containing increasing concentrations of SAAP-148 or halicin, 50 μL of pooled human plasma, pooled human urine or PBS, and finally, 20 μL of the bacterial suspension were pipetted into wells of a polypropylene V-shape microplate (Greiner BioOne, Frickenhausen, Germany). After incubation for 4 h at 37 °C under rotation at 200 rpm, the number of viable bacteria was assessed microbiologically. Results are expressed as lethal concentration (LC) 99.9, i.e., the lowest concentration of the agent that killed 99.9% of the inoculum.

### 4.4. Anti-Biofilm Assay

Bacteria were diluted in BHI (Brain Heart Infusion broth, Oxoid) for 7 days biofilms and in BM2 medium (prepared as previously described [47]) for 24 h biofilms. Briefly, 100 μL of a suspension of log-phase bacteria (1 × 10^7^ CFU/mL) was added to each well of a polypropylene flat bottom microplate (Greiner BioOne, Germany) and incubated for 24 h or 7 days at 37 °C. Next, the planktonic bacteria were removed from the wells and the latter was washed twice with 100 μL of PBS to remove the remainder of the non-adherent cells. The biofilms were subsequently exposed to increasing concentrations of the peptide or halicin in PBS (both at range 0–204.8 μM) for 4 and 24 h at 37 °C. Plates with 24-h biofilms were sealed with non-breathable plastic film sealers (Amplistar adhesive plate sealers, Westburg), while plates with mature biofilms were sealed with breathable Ryon film sealers (VWR European). Medium controls were used to monitor possible contamination. Finally, the biofilms were washed once with PBS, the bacteria were harvested in 100 µL PBS by sonication (Branson 1800, 10 min), and the number of viable bacteria was assessed microbiologically. Results are expressed as biofilm-inhibiting concentration (BIC) 99, i.e., the lowest concentration of the agents resulting in 99% reduction in bacterial counts.

### 4.5. Hemolysis Assay

Citrated whole human blood from healthy volunteers was centrifuged at 3000 rpm to pellet the erythrocytes and washed three times in PBS before preparing a 2% erythrocyte suspension in PBS. Subsequently, 25 μL of PBS containing increasing concentrations of SAAP-148 peptide or halicin were mixed with 50 μL of pooled human plasma or PBS and 25 μL of 2% human erythrocytes in wells of a polypropylene V-shaped microplate. A 5% (*v*/*v*) Triton-X solution in PBS was included as positive control and PBS as a negative control. The plate was incubated for 1 h at 37 °C and 5% CO_2_, after which the erythrocytes were pelleted by centrifugation for 3 min at 1200 rpm. The supernatant was transferred to a 96-wells flat-bottom plate and the optical density was measured at 415 nm. Results are expressed as IC_50_, i.e., the concentration of the agent inducing 50% reduction of cytotoxicity. Non-linear regression curves with bottom and top restrictions at 0 and 100% were fit for each individual experiment to determine the medians (and ranges) of the IC_50_ values.

### 4.6. Assays for Cytotoxicity Using Human Primary Skin Fibroblasts and Human RT-4 Urothelial Cells

Human primary skin fibroblasts (kindly provided by M.H. Rietveld, Department of Dermatology, LUMC) were cultured in culture flasks using DMEM supplemented with 1% (*v*/*v*) GlutaMAX™, 1% (*v*/*v*) pen/strep and 5% (*v*/*v*) FCS. Next, fibroblasts were harvested using 0.05% trypsin-EDTA, washed and resuspended to 2 × 10^5^ cells/mL and finally 20,000 cells were seeded in 96-wells culture plates. These cells formed monolayers overnight at 37 °C and 5% CO_2_. Human urothelial RT-4 cells were cultured in culture flasks using McCoy’s medium supplemented with GlutaMAX™, 1% (*v*/*v*) pen/strep and 10% (*v*/*v*) FCS. Cells were harvested and resuspended to 4 × 10^5^ cells/mL and finally, 40,000 cells were seeded in 96-wells culture plates. These cells formed monolayers upon 36–48 h of incubation at 37 °C and 5% CO_2_. Thereafter, the monolayers were exposed for 4 h or 24 h to increasing concentrations of SAAP-148 or halicin in DMEM supplemented with GlutaMAX™, pen/strep and 0.5% (*v*/*v*) human serum. For RT-4 cells, their culture medium was used for the assay. 1% (*v*/*v*) Triton-X was used as positive control and medium as negative control. LDH release from dead cells into the supernatants was detected by the Cytotoxicity Detection Kit (Roche, Cat. No. 1644793) and the metabolic activity of the cells was assessed using the WST-1 reagent (Cell proliferation reagent WST-1; Roche, Cat. No. 11644807001)), both according to manufacturer’s instructions. Results are expressed as IC_50_, i.e., the concentration of the agent inducing 50% reduction of cytotoxicity or metabolic activity. Non-linear regression curves with bottom and top restrictions at 0 and 100% were fit for each individual experiment to determine the medians (and ranges) of the IC_50_ values.

### 4.7. Checkerboard Assay

A checkerboard assay was performed against multiple strains of AMR *E. coli*, *A. baumannii* and *S. aureus*. For this purpose, mid-log phase bacteria were resuspended in RPMI 1640 with 20 mM HEPES and L-glutamine and without sodium bicarbonate (Sigma Life Science, Saint Louis, MO, USA), further referred to as RPMI mod, to a concentration of 4 × 10^6^ CFU/mL. Subsequently, 100 µL of increasing concentrations of halicin, 50 µL of increasing concentrations of SAAP-148 and 50 µL of bacterial suspension (all in RPMI mod) were mixed in the wells of a 96-well flat-bottom polypropylene plate (Greiner BioOne). The plates were covered with a breathable seal and incubated for 18–20 h at 37 °C in a humidified environment. After incubation overnight, the plates were shaken at 1000 rpm for 10 s and the optical density at 450 nm was measured.

The fractional inhibitory concentration index (FICI) scores were calculated using the following formula:

(1) $$FICI\;score=\frac{MIC_{SAAP-148,\;combination}}{MIC_{SAAP-148,\;alone}}+\frac{MIC_{halicin,\;combination}}{MIC_{halicin,\;alone}}$$

A FICI score of ≤0.5 indicates synergism, a FICI score between 0.5 and 1 represents an additive effect, a FICI score between 1 and 4 demonstrates no interaction between the agents and a FICI score > 4 implies antagonism [48].

### 4.8. Biofilm Model on Elastomer Disks

Mid-log phase bacteria were resuspended in BHI to a concentration of 1 × 10^7^ CFU/mL. Subsequently, sterile silicone elastomer disks (punched with biopsy punch Ø 4 mm, Stiefel, #2957 out of a silicone elastomer sheet, SI303060, sheet 600 × 600 mm, Goodfellow Cambridge Ltd., Huntingdon, UK #572-667-36) and 100 µL of bacterial suspension were added to the wells of a flat-bottom 96-well polystyrene plate. Plates were sealed with breathable Ryon film sealers and incubated for 24 h at 37 °C in humid conditions. After 24 h, disks with adhered biofilm were washed by transferring them to a flat-bottom 96-wells polystyrene plate containing 200 µL PBS/well and shaking the plate for 10 s at 600 rpm. Subsequently, the disks with adhered biofilm were transferred to a flat-bottom 96-wells polypropylene plate and exposed to 100 µL of PBS containing increasing concentrations of SAAP-148 and/or halicin for 4 h at 37 °C. Finally, the biofilms were washed again with PBS, the bacteria were harvested by 10 min sonication and the number of viable bacteria was assessed microbiologically. Results are expressed as individual values and medians of at least three individual measurements performed in duplicate.

### 4.9. 3D Bladder Infection Model

RT-4 cells, a kind gift from Dr G van der Pluijm (Department of Urology, LUMC, Leiden), were grown in culture flasks using McCoy’s medium supplemented with glutamax, 1% (*v*/*v*) pen/strep (Gibco, Cat. No. 15140122) and 10% (*v*/*v*) fetal calf serum (FCS, Corning). After several washes, the RT-4 cells were harvested using 1 mL of 0.05% trypsin-EDTA (Gibco; phenol red), washed and grown on Thincerts (pore size 0.4 µm, Greiner BioOne) in a 12-wells deep well plate (Greiner BioOne) at a concentration of 1 × 10^6^ cells/well. The cells were cultured in McCoy culture medium for approximately three weeks, while changing the medium regularly, resulting in a 3D urothelial model. One day before infection, the culture medium below the models was replaced for culture medium without antibiotics and above the models, for 50% pooled urine in PBS. On the day of infection, the models were moved to a 12-well cell culture plate (Costar, Corning Inc., Kennebunk, ME, USA) and infected with *E. coli* LUH15108 in 50% urine at a concentration of 1 × 10^4^ CFU/model for 1 h at 37 °C and 5% CO_2_. After infection, the bacterial suspension was removed, the models were washed with PBS and then exposed to SAAP-148 or halicin at the desired concentrations in 50% urine for 4 and 24 h, after which the supernatants (bacteria in luminal compartment) were stored on ice, while the models (bacteria in cellular compartment) were homogenized using a bead-beater (Bertin technologies, Precellys 24 lysis and homogenization, 5000 rpm, 3 × 10 s, 10 s pause) and both fractions were serial diluted and plated on Mueller–Hinton (MH) plates overnight at 37 °C. Results are expressed as individual values and medians of at least three individual measurements performed in duplicate.

### 4.10. 3D Human Epidermal Infection Model

Human skin equivalent (HSEs) were cultured according to a modification of a previously described method [49]. Human keratinocytes of the Ker-CT cell line (ATCC^®^ CRL-4048™) were cultured under submerged conditions in keratinocyte serum-free medium (KSFM; Gibco, #17005-034) supplemented with bovine pituitary extract (BPE; Gibco, #37000-014), human recombinant epidermal growth factor (EGF; Gibco, #37000-013), 0.3M CaCl_2_ (Merck, #2381), and 1% (*v*/*v*) penicillin-streptomycin (Gibco, #15140-122). The keratinocytes were harvested using trypsin-EDTA, washed and grown onto ThinCert transwells (pore size 0.4 µm, Greiner BioOne, #665641) in 12-well culture plates (Costar, Corning Inc.) using 2 × 10^5^ cells/well in 400 µL of supplemented DermaLife K medium (Lifeline Cell Technology, Frederick, USA, #LL-0007) above the transwell with 1 mL of medium below the well. When the cells reached confluency (after 2 days), DermaLife K medium was replaced above and below the transwell with 50% K0 medium [three parts DMEM (Gibco, #31966), one part Ham’s F12 medium (Gibco, # 21765), 200 ng/mL hydrocortisone (Sigma-Aldrich, #H0888), 2 μM isoproterenol (Sigma-Aldrich, #1351005), 500 ng/mL bovine insulin (Sigma-Aldrich, #16634), 53 nM selenious acid (Sigma-Aldrich, #229857), 10 mM L-serine (Sigma-Aldrich, #PHR1103), 10 μM L-carnitine (Sigma-Aldrich, #C0283)] and 50% CNT-Prime 3D Barrier Medium (CELLnTEC, #CnT-PR-3D) supplemented with a lipid mixture (1:1:1) containing 15 μM linoleic acid (Sigma-Aldrich, #L8134), 25 μM palmitic acid (Sigma-Aldrich, #P0500) and 7 μM arachidonic acid (Sigma-Aldrich, #A3611), in 80 mg/mL bovine serum albumin (Roche Diagnostics #10735094001). Models were incubated overnight, and the next day, the models were air-exposed by aspirating the medium on top of the filter. The air-exposed models were cultured for approximately 10 days while changing the medium regularly with K0/CNT-medium as detailed above, with a lipid mixture (2:1:1) containing 30 µM linoleic acid instead. At least two days before infection, the culture medium was replaced by culture medium without antibiotics. The HSEs were infected with MRSA LUH14616 at a concentration of 1 × 10^5^ CFU/model for 1 h at 37 °C and 5% CO_2_. After infection, the bacterial suspension was removed, the cells were washed with PBS and the models were treated with SAAP-148 or halicin at the desired concentrations for 4 h after which the supernatants (non-adherent bacteria) were stored on ice, while the models (adherent bacteria) were homogenized using a bead-beater, and both fractions were serial diluted and plated on MH plates overnight at 37 °C. Results are expressed as individual values and medians of three individual measurements performed in duplicate.

### 4.11. Statistics

Differences between two groups (SAAP-148, halicin and/or combinations of these agents) in human bladder infection and human epidermal infection models were evaluated by a Kruskal–Wallis test, followed by a Mann–Whitney rank sum test using Graphpad Prism software version 6.0 (Graph Pad Software, San Diego, CA, USA). Differences were considered statistically significant when *p* < 0.05.

## Figures and Tables

**Table 1:** In vitro killing of planktonic AMR *E. coli*, *A. baumannii* and *S. aureus* by SAAP-148 and halicin.

Species	Strain	LC_99.9_ SAAP-148 (µM)			LC_99.9_ Halicin (µM)			
		PBS	50% Urine/Plasma		PBS		50% Urine/Plasma	
***E. coli***	LUH15174	**0.8**	**1.6**	(0.8–1.6)	**12.8**	(6.4–51.2)	**51.2**	(6.4–>51.2)
***A. baumannii***	RUH875	**1.6**	**6.4**		**25.6**	(25.6–51.2)	**>102.4**	
***S. aureus***	LUH14616	**1.6**	**12.8**		**102.4**	(102.4–>102.4)	**>204.8**	

**Table 2:** In vitro killing of biofilm-residing AMR *E. coli*, *A. baumannii* and *S. aureus* by SAAP-148 and halicin.

Species	Strain	BIC_99_ SAAP-148 (µM)				BIC_99_ Halicin (µM)			
		24 h Biofilm		7 Days Biofilm		24 h Biofilm		7 Days Biofilm	
		4 h	24 h	4 h	24 h	4 h	24 h	4 h	24 h
***E. coli***	LUH15174	**204.8**	**68.3**	**204.8**	**68.3**	**204.8**	**68.3**	**204.8**	**≤22.8**
		(68.3–204.8)	(68.3–204.8)	(204.8–>204.8)	(≤22.8–68.3)	-	(68.3–204.8)	-	(≤22.8–68.3)
***A. baumannii***	RUH875	**68.3**	**204.8**	**68.3**	**68.3**	**204.8**	**>204.8**	**204.8**	**68.3**
		-	-	-	-	-	(204.8–>204.8)	-	(68.3–204.8)
***S. aureus***	LUH14616	**204.8**	**204.8**	**68.3**	**68.3**	**>204.8**	**68.3**	**>204.8**	**68.3**
		(68.3–204.8)	-	-	-	-	(68.3–204.8)	-	-

## Data Availability

The data presented in this study are available within the article or are available on request from the corresponding author.
